# Editorial

**Published:** 1898-02

**Authors:** 


### Editorial.

The conviction of the editor of an Iowa medical publication on the charge of sending obscene literature though the mail, the ground for the charge being the reproduction of some blackboard vulgarity intended by the students of a medical college for the benefit of the "co-eds," arouses the question of how far a scientific publication may dare to go without overstepping legitimate rights. That some medical journals take advantage of the axiom that nothing scientific is base, is at times painfully evident both as to matter and illustration, yet the truth of the axiom, though strained, it is not broken. Both science and art live in the upper air where the contaminating influence of conventionality and popular prejudice or prepossession does not reach. In both, the human body undergoes an apotheosis in which exaltation the baser passions and attributes of the flesh fall away to discover the perfect man.

With such conceptions in view, how detestable are the perversions, the degradations whereby both are made the shields behind which obscenity and pruriency crouch. Science is made the unwilling cover of all manner of filth. Some years ago while in Paris, the writer was decoyed into purchasing a book purporting to be a study of some morbid psychological phenomena, but found out after reading the first few pages that it was no more than the biography of a cocotte, beginning with her seduction by her employer and ending by her establishing herself as the landlady of a *maison de tolerance*. The psychological feature consisted in the discussion of sexual perversion with a fidelity to nature and a wealth of detail which left nothing wanting to show the vile purpose of the "study." Another instance in point, but rendered more heinous in offense by the fact that it is a work of imagination, is Belot's "Mlle. Girard, ma femme," a romance of Sapphism.

Similar *quasi* scientific romances have been attempted from time to time in English, but lacking the delicacy of the Gallic touch, the musk perfume of French naughtiness, is converted into a strong fecal odor. The reading public did not take kindly to them, and they have ceased for the time at any rate to appear, be it said to the eternal credit of the American people.

With such equivocal productions the medical profession has nothing to do—the bond of union between this "science" and that which animates the study of disease and its cure is too feeble to resist any effort to separate them. But it is to be regretted that while we seek to pluck out the mote from another's eye, we ignore the beam that is in our own. Some time ago there appeared in a Western medical contemporary an article on sexual perversion which was so vivid in its depiction of the several types, that the editor wisely withdrew though at the cost of leaving it suspended on a "to be continued." The editor realized in time that the food was too coarse for the average medical stomach, and that he was liable to be inundated in its too speedy, projectile return. It must be said, in justice to the editor, that there is a bare possibility of his never having read it. Editors have been known to do this.

The risque quality in illustration is beginning to pervade medical as well as lay literature. We have become hardened to the sight of well-filled suits of union underwear: young ladies in various states of *dishabille* advertising not only their charms but some soap or confection or toilet accessory; but they have been hitherto confined to the advertising pages (often the most interesting) of the cheap illustrated magazines. Apart from an occasional poor and dim wood cut of some female uncomfortably spread upon a surgical chair to further the sale of the latter, and a tripodal, bodiless monster with a bubo in the lonesome groin and something else the matter with each leg, medical journals have been rather free from spectacular sin. But one which appeals to what has been termed the "submerged third," has a small cut, among its advertisements, evidently a half-tone photograph of a buxom young female clad in that, as Mark Twain would say, "modification of the highland costume," that is, nit—Mother Eve, the only woman who was never influenced by the prejudices of fashion, contented to cover her nakedness with fig leaves. The young person in the cut has substituted for fig-leaves a "natural body brace," to serve the same purpose as the leaves, but what a difference between the insouciance of our common mother and the brazen show of this nineteenth century Phryner.

The pity of it is not that this maid should unmask her charms so prodigally, but that justification for it should be sought in that she advertised something to relieve disease and pain, that an unhallowed use was made of a great and pure principle, and that a medical journal, whose mission it should be to labor for the cause of good morals and social purity, should allow it.

We live in an age of increasing tension. Men are throwing off restraint of religion, of morals, of manners, and women are straining at the leash. It is a new condition and not a repetition of history. When was there ever such unbridled liberty of the press, of literature, of the stage? A spade was called a spade in the days of Tristram Shandy, Peregrine Pickle and Squire Weston; but it was the men who called it a spade, not the women. Who is itthat speaks more broadly now.

It is useless to enter further into the subject. The way is perfectly clear for us of the medical profession and medical journalists. We must ever be the champions of purity and our own conscience is the guide and censor; and we need no other standard.

